# Development of a checklist for systematic screening of precipitating factors in older patients admitted to hospital with delirium

**DOI:** 10.1007/s41999-025-01191-2

**Published:** 2025-04-20

**Authors:** Carmelo Lafuente-Lafuente, Evany Heinichen Candia, Eric Pautas, Yonathan Freund, Bruno Oquendo, Joël Belmin, The Improving Management of Delirium in Older Patients (IMDO) Investigators, Laura Arfaoui, Jacques Boddaert, Quentin Delannoy, Zineb Kacher, Christel Oasi, Elena Paillaud, Sylvie Pariel, Marc Verny

**Affiliations:** 1 Hôpital Charles Foix, Service de Gériatrie à orientation Cardiologique et Neurologique, Sorbonne Université, APHP, 7 avenue de la République, 94205 Ivry-sur-Seine, France; 2 Clinical Epidemiology and Ageing (CEpiA) Team, Université Paris Est Créteil, INSERM, IRMB, 94000 Créteil, France; 3 Facultad de Ciencias de la Salud, Universidad Católica, Asunción, Paraguay; 4 Hôpital Charles Foix, Service de Gériatrie Aigue Polyvalente, Sorbonne Université, APHP, 94200 Ivry-sur-Seine, France; 5 Hôpitaux Universitaires Pitié-Salpêtrière, Service d’Accueil des Urgences, Sorbonne Université, APHP, 75013 Paris, France; 6 Laboratoire LIMICS, Sorbonne Université, Paris, France

**Keywords:** Delirium, Older people, Etiology, Precipitating factors, Clinical diagnosis, Checklist

## Abstract

**Objectives:**

Causes of delirium are often multiple in older people and a significant number of them are missed at the initial assessment. We wanted to develop a checklist to help clinicians to systematically screen for the most frequent and important causes of delirium in this population.

**Methods:**

Three investigators, based on previous studies, drafted three possible checklist base models, comprising 18–29 items. A panel of 12 experts voted to choose one of the models, and then followed a modified Delphi consensus method to review each item and further develop the checklist. The consensus checklist was then tested in a small group of patients. The target population was older patients with delirium arriving to the emergency department or to a medical hospital ward.

**Results:**

The longest of the three drafts was unanimously chosen by the panel. After three rounds, a consensus was reached and a final checklist agreed. It is composed by 27 items organized in five groups of causes: infections, hydro-electrolytic disturbances, drugs, acute neurological conditions, and other acute diseases. A pilot study conducted by 15 physicians in 21 consecutive patients showed that the checklist was easy to complete, quick (mean 4 min) and most clinicians, though it helped them through the initial diagnostic work.

**Conclusions:**

We have developed and pilot tested a checklist for screening for acute precipitants of delirium in older patients, aimed to facilitate early recognition and treatment of the multiple causes that often coexists in this population.

**Supplementary Information:**

The online version contains supplementary material available at 10.1007/s41999-025-01191-2.

## Key summary points

**Aim:**

To develop a checklist to help clinicians screen for acute precipitants of delirium in older patients, as some precipitants are frequently underdiagnosed initially.

**Findings:**

A panel of experts developed a checklist for this purpose, composed of 27 items organized in 5 groups of causes. In a pilot study, this checklist was easy to do, took only a few minutes, and helped clinicians to identify causes of delirium.

**Message:**

We have developed a checklist to facilitate the search for acute causes of delirium in older patients and reduce the risk of overlooking some of them.

**Supplementary Information:**

The online version contains supplementary material available at 10.1007/s41999-025-01191-2.

## Introduction

Delirium, or acute confusional state, is a clinical syndrome characterized by disorientation, impaired attention, abnormal level of consciousness, and disorganized thinking, all of them of acute onset and usually fluctuating [1, 2]. It is very frequent in older patients with acute conditions admitted to emergency departments (12–18% of older patients) or to hospital, either in surgical or medical wards (14–25% of older patients) [1, 3–5]. Although usually reversible, delirium is a severe condition and is associated with higher short-term mortality and impaired long-term cognitive outcomes [6–8].

Delirium can be precipitated by many diverse acute organic conditions. In a previous study on 207 consecutive older patients admitted to hospital with delirium, we found infections, hydro-electrolytic disorders, adverse drug effects, and acute neurological conditions as the most frequent causes precipitating delirium [9]. Other studies have also found acute renal failure, intracranial hemorrhage, cardiorespiratory acute diseases, pain, physical restrain, and invasive catheters and procedures as frequent precipitants of delirium [1, 10]

Non-pharmacological multidimensional interventions are effective in preventing delirium in adults admitted to hospital by any reason [11]. Once delirium occurs, however, one of the most important action in its management is to quickly identify and treat all acute conditions that have precipitated delirium [12–14]. Unfortunately, delirium is often underdiagnosed, especially in hypoactive, somnolent patients: in as much as 50% of patients in emergency department and 22% in medicine wards [15, 16]. Even when recognized, one or more acute precipitants contributing to delirium are frequently missed initially. In our previous study, we found that most patients had several concomitant causes (mean 2.3) and that one or more acute precipitants of delirium was missed at the initial assessment in about 25% of patients [9]. Several mnemonics and short lists have been proposed to help identifying acute precipitants of delirium, usually integrated in general pathways for managing delirium [14, 17]. We wondered if a more detailed and effective checklist could be developed, as a separate tool.

Therefore, we decided to develop a comprehensive checklist to help clinicians to systematically search for the most common and important causes of delirium in older persons. The objective was to minimize the number of treatable and reversible causes that might be missed at the first assessment of the patient.

## Methods

### Objective

To develop a checklist for systematically screening for the most frequent and important acute conditions precipitating delirium in older patients in medical settings (emergency department, hospital medical wards, and nursing homes). We did not consider delirium happening after surgery.

### Study design

Expert consensus, using a modified Delphi method, completed with pilot testing of the final checklist in a small group of patients.

### Checklist conception

We conceived the checklist mainly as a memory aid, not a decision-making aid, for use in real time, just after a clinical suspicion or a formal diagnosis of delirium in an older patient. The target population was older patients admitted to the emergency department or hospital acute wards, in medical settings. We wanted the checklist to be independent but able to be coupled with other tools employed for the screening or the diagnostic of delirium, like the 4 ‘A’s Test (4AT) or the Confusion Assessment Method (CAM), if already commonly employed in that department.

### Experts’ panel

Twelve physicians with active clinical practice and experience in the management of delirium, comprising specialists in geriatrics, neurogeriatrics, internal medicine, and emergency medicine, from several university hospitals in Paris area, France.

### Choice of an initial checklist model

Three investigators (CLL, EH, and ZK), based in a review of the literature, detailed data from a previous study we conducted [9] and recommended practices for designing checklists in medicine [18, 19], elaborated three draft checklists differing in composition and extension, as possible initial working models.

The shortest draft checklist selected only the most frequent causes and comprised 18 items, organized in 4 sections. The longest one comprised 28 items, organized in 5 sections. The 3 draft checklists (in French) are displayed in the Appendix (Figures S1–S3).

The panel experts voted then to choose one between those three drafts, as the base model to start working with.

### Refining the checklist

The selected base model checklist was then reviewed by the experts in successive rounds. Each time, each expert independently reviewed all items composing the checklist and, for each one, they stated if they agreed with the item as it was, if they agreed with but though it should be modified, or if they disagreed and though it should be deleted. Additionally, they reviewed the distribution of the items and overall organization of the checklist, and stated if they would introduce any modification or to add any item.

**Fig. 1:**
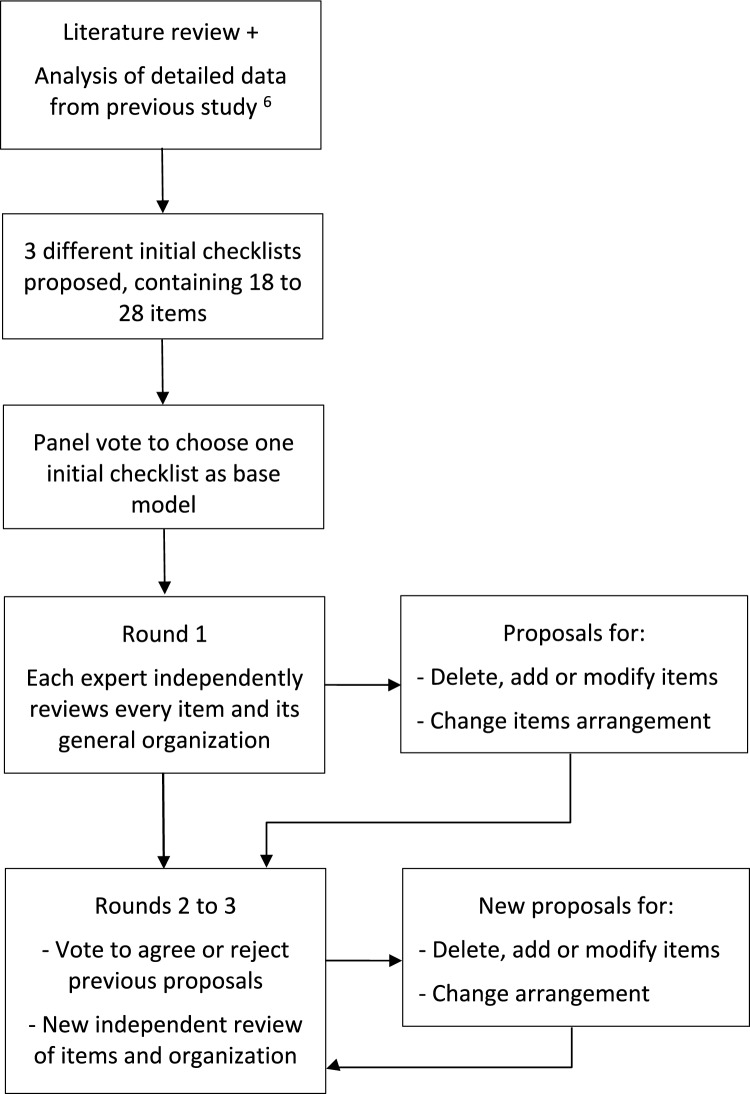
Methods followed to develop the checklist

Modifications suggested were then presented in the checklist and voted on. They were retained if 65% or more of experts agreed (this is, 8 experts out of the 12) and rejected otherwise. Three rounds were planned (Fig. 1).

### Pilot testing

The final checklist was then tested in real conditions in a small group of 15–20 consecutive older patients admitted to the Charles Foix hospital Geriatrics Department with a diagnosis of delirium, using the CAM diagnostic criteria [20]. Clinicians using the checklist answered a short questionary after using the checklist regarding its feasibility, time needed for completion, and perceived usefulness. The study was approved by the corresponding regional ethics committee (*Comité de Protection de Personnes d’Île-de-France II*, study number 21.00279.000003 RIPH3 HPS). Informed consent was obtained from all participants included.

## Results

All panel experts unanimously chose the longest of the three proposed drafts as the initial base mode on which to build the checklist. During the developing process, 41 modifications were proposed by the experts: 22 in the first round, 11 in the second round, and 8 in the third round. Nine proposals were rejected, and all the others were integrated in the checklist.

After three rounds, a large consensus was achieved and no new modifications of any item in the checklist were suggested. Items added, deleted, and amendments introduced in the successive rounds are presented in Table 1. The most significant changes introduced in the initial model were: (a) completely rewriting the items under "acute neurologic disorders"; (b) the addition on the back of the page, as optional additional information, of the Confusion Assessment Method (CAM) diagnostic criteria for delirium [20] and a more detailed list of predisposing and precipitating causes; (c) adding a "not available" checkbox to all items concerning complementary tests (radiology and biology); and, (d) expanding the list of pharmacological classes under "Drugs". Otherwise, some minor modifications in the wording and disposition of elements on the sheet were introduced.

**Table 1:** Modifications introduced in the checklist in the successive rounds

Category	First round	Agreed	Second round	Agreed	Third round	Agreed
General	Delete "Yes/No" boxes, put just one box to check	No				
	Delete question marks	Yes				
	Add "*Not available*" box on biology and imaging items	Yes				
	Add list of predisposing and precipitating factors on the back	Yes	Delete list of predisposing factors on the back	No		
	Add delirium diagnostic criteria on the back	Yes	Put delirium diagnostic criteria on the main page	No		
Infections	Describe “*respiratory symptoms*”	Yes				
	Define values for “*elevated*” procalcitonin	Yes	Delete procalcitonin	Yes		
			State “*Procalcitonin* > *0.2 ng/ml*”	No		
Biology	Do not ask for specific values of creatinine/natremia/calcemia	Yes	Delete calcemia	No	Delete calcemia	Yes
			Define values for hypoglycaemia	Yes	Say “*Abnormal glycaemia*”. Do not state values	Yes
Drugs	Add “*Recent introduction or change*”	Yes				
	Add “*Antipsychotics (neuroleptics or atypical)*”	Yes			Add “*Anti-cholinergic drugs*”	No
	Add “*Sedative drugs*”	No			Add examples of narcotics	Yes
Neurologic disorders	Add “*Acute*” to neurologic disorders	Yes				
	Put list from M1 model instead	Yes				
Neurologic disorders (continue)	Make brain CT/IRM item as a question	Yes				
	Say “*Head trauma without bleeding complications*”	No				
			Say “*Coma and history of epilepsy*” instead of “*Epilepsy*”	No	Say “*History of epilepsy*”	Yes
			Say “*Coma and focal signs*”	No	Delete “*Coma*”	Yes
Other acute conditions	Add "*Acute*" to heart and respiratory failure	Yes				
	Add “Alcohol: acute intoxication or withdrawal”	Yes				
	Add “*Faecaloma*”	No				
	Add “*Urinary retention*”	No				

While most suggestions easily obtained a large consensus, some points were more controversial and needed more debate to reach an agreement, as for example adding or not a question about calcemia (finally deleted, as not a frequent cause), keeping or deleting "coma" (finally suppressed, as considered a separate clinical situation) or adding "urinary retention" and "fecaloma" (finally not added, because, even if traditionally teached as a causes of delirium, we found very limited supporting evidence, limited to a few case reports [21]).

Asked about their opinion on the usefulness and feasibility of the checklist in clinical practice, 2 of the 13 experts thought that the checklist reflected very closely their clinical practice and would apport no additional value. Five experts, conversely, thought that it would be useful to routinely use it in older patients with delirium.

### Pilot study

The resulting checklist was then tested by 19 different physicians in 21 consecutive patients with delirium admitted to two acute geriatric units at Charles Foix hospital. Table 2 lists the characteristics of patients and results obtained using the checklist. Patients’ mean age was 84 years (range 67–99) and 12 (57%) were women. All patients but one lived at home. Eleven patients (52%) had one or more apparent pre-existing predisposing factor, a history of cognitive disorder being the most frequent. Based on the diagnostics stablished at the end of their stay in acute care, 1.5 acute precipitants of delirium per patient were found on average. Infections (4 COVID-19, 3 pneumonia, and 3 urinary tract infections), hydro-electrolytic disorders, and drugs were the most common acute precipitants.

**Table 2:** Pilot study : characteristics of patients and physicians, results

Patients n = 21	N (%) (Unless otherwise specified)
Age, years (mean (SD))	84 (9)
Women	12 (57%)
**Previous history of:**	
Cognitive disorder	8 (38%)
Stroke or transient ischemic attack	3 (14%)
Parkinson disease	3 (14%)
Depression	4 (19%)
**Acute precipitants of delirium found:**	
Infections	10 (48%)
Dehydration and/or hyponatremia	6 (29%)
Acute heart or respiratory failure	5 (24%)
Drugs	5 (24%)
Stroke, ischemic or hemorrhagic	3 (14%)
Other	3 (14%)
Number of precipitants by patient (mean (SD))	1.54 (0.2)
**Physicians using the checklist**	n = 19
Senior doctor	6
Resident	13
Estimated time for completing the checklist, minutes (mean (SD))	4 (2.6)
Felt that the checklist allowed him/her to found additional precipitants	5 (26%)
**Declared using the checklist was:**	
Easy	17 (90%)
Neither easy nor difficult	1 (5%)
Difficult	1 (5%)
**Declared they will use the checklist:**	
Frequently	10 (53%)
From time to time	5 (26%)
Rarely	4 (21%)

Mean time participating doctors estimated they had taken for completing the checklist was 4 min (range 2–10). In all cases but 2, clinicians thought the checklist was easy or very easy to fulfill. In 5 of the 21 patients, the clinician believed that the checklist had allowed them to find a cause of delirium that otherwise they had probably ignored. Ten of the 15 physicians stated that they would use the checklist frequently in their usual practice. Three physicians, however, though that they had already integrated most items in their current practice and that the checklist did not apport them any additional value. Asked, none of the participating clinicians suggested any further modification in the checklist.

### Final checklist

The checklist finally developed is structured in five sections, each comprising a list of items: (1) infections, (2) hydro-electrolytic disorders, (3) drugs, (4) acute neurological disorders, and (5) other acute conditions. As an option, it includes on the back additional information about signs and situations when to suspect delirium, a reminder of the CAM diagnostic criteria, and a more comprehensive list of predisposing and precipitating factors of delirium.

The original French version as well as an English translation can be found in the Appendix, or downloaded from https://osf.io/mrpc5/ (10.17605/OSF.IO/MRPC5). An interactive version (French only) is also available at https://adrem-med.org/score.php?id=38.

## Discussion

In this work, we developed, with the help of a panel of expert geriatricians, emergency physicians, neurologists, and internists, a checklist to help systematically search for the most important acute conditions precipitating delirium in older patients in medical settings. The resulting *Improving Management of Delirium in Older patients* (IMDO) checklist includes 27 items grouped in 5 categories: infections, hydro-electrolytic disturbances, drugs, acute neurological disorders, and other acute conditions. In a pilot study, the checklist was easy and swift to complete. More importantly, the use of the checklist prompted the clinician to consider in some patients acute precipitants of delirium that otherwise he might had overlooked. Prompt treatment of those precipitants is the mainstay of the management of delirium, the most effective measure for shortening it, and the ultimate aim of this checklist [13, 14].

This checklist is not exhaustive; this was not the objective. More than 100 acute conditions have been reported precipitating delirium in older patients [22]. A comprehensive checklist would be excessively long, time-consuming and not efficient in real practice. Rather, the checklist intends to focus on the most common causes of delirium, particularly on those most frequently missed and on those that, left untreated, could aggravate patients’ evolution and prognosis. Nevertheless, the practitioner shall keep in mind that infrequent causes of delirium, not included in this checklist, may exist.

Several global frameworks for managing delirium integrate checklists or prompts to help clinicians search for acute precipitants, like the TIME bundle or the more recent Delirium Delphi Algorithms [14, 17, 23]. While undoubtedly useful, the lists included in those tools comprise generally a few items (5–10) which are large in scope, requiring for example to “look for symptoms/signs of infection” or “review drugs and other intoxicants”. The checklist here developed is more detailed, it expands each main group of precipitants into several items, and each item is very specific. In any case, the aim of the IMDO checklist is not to replace the existing tools for delirium nor to cover all that is needed to manage delirium. The IMDO checklist does not tell, for example, what to do after diagnosing a given precipitant, just “to rapidly initiate appropriate treatment”. Its objective is to complement other tools on the search for precipitants. Thus, it can be easily integrated into general frameworks for delirium (TIME, Delphi), or combined with other tools designed for screening for or diagnosing delirium, as the 4 ‘A’s Test (4AT), the Confusion Assessment Method (CAM) or the Nursing Delirium Screening Scale (Nu-DESC) [24–26].

There is not, to the best of our knowledge, any generally agreed method for developing checklists in medicine [19, 27]. Problems faced in clinical medicine are by nature more varied, less reproductible and harder to systematize than problems encountered in other domains where checklist are commonly employed, like aviation or industry. Weisser et al. took inspiration from the process followed in aviation and adapted the Plan-Do-Study-Act (PDSA) model there employed to create the WHO Surgical Safety Checklist instead 18]. Many checklists, however, have been elaborated without following a clear methodology 19, 28]. We have followed the framework proposed by Burian et al. for developing checklists in medicine, which comprises five stages covering the entire checklist life cycle: (1) conception, (2) determination of content and design, (3) testing and validation, (4) induction, training, and implementation, and (5) ongoing evaluation and revision [19]. The work we report here corresponds to the first two-to-three stages of this framework, so it would need further validation and could evolve in the future by integrating feedback from experience.

Introducing a checklist in practice can also have educational value. Delirium in older people and its consequences are often poorly understood by healthcare professionals and frequently overlooked 29, 30]. Presenting the checklist, its rationality and objective, together with the additional information included on its back, may contribute to improve the knowledge of healthcare professionals about delirium in older people, thus improving its management.

A limitation of this checklist is that it has not been developed for younger patients, nor for patients with surgical conditions, following surgery or in intensive care units. While many frequent precipitants of delirium are shared, this checklist might not include some causes of delirium that are frequent in surgical or intensive care settings. A second limitation is that we did not involve nurses or pharmacists in the development of the checklist, professionals who may contribute to the diagnostic of acute precipitants of delirium in some patients. Finally, as already discussed, this checklist has been tested only in a small group of older patients. Its feasibility, reproducibility, and diagnostic sensibility would need to be tested in a larger number of patients in real clinical practice. We are currently conducting a clinical study to this end.

In conclusion, a panel of experts developed in this work a checklist, comprising 27 items grouped in five categories, to help clinicians to systematically search for the most frequent and important acute causes of delirium in older people presenting with this condition at the emergency department and in medical wards. A pilot test in a small number of patients showed that the checklist is easy to use, takes only a few minutes to complete and that, at least in some patients, helps clinicians to identify a cause of delirium that otherwise they would have probably overlooked. The ultimate goal of the checklist is to detect and treat as early as possible all acute causes of delirium existing in each patient.

Further studies, involving a greater number of patients, will be needed to validate the reproducibility and the sensibility of the IMDO checklist to detect causes of delirium in this population.

## Supplementary Information

Below is the link to the electronic supplementary material.

Supplementary file1 (DOCX 316 KB)

## Data Availability

The final checklist (French and English) and full dataset of the pilot study are available from the Open Science Framework, 10.17605/OSF.IO/MRPC5.
