# Supplementary material for: Development of a checklist for systematic screening of precipitating factors in older patients admitted to hospital with delirium
Source: Eur Geriatr Med. 2025 Apr 20;16(3):861–9. doi: 10.1007/s41999-025-01191-2 (PMC12174225; doi:10.1007/s41999-025-01191-2)
Supplement: Supplementary file 1 — Supplementary file1 (DOCX 316 KB) [file 41999_2025_1191_MOESM1_ESM.docx]

Supplementary Data

# Development of a checklist for systematic screening of precipitating factors in older patients admitted to hospital with delirium

Lafuente-Lafuente C, Heinichen Candia E, Pautas E, Freund Y, Oquendo B, Belmin J and the Improving Management of Delirium in Older patients (IMDO) Investigators.

# Figure S1. Draft base models developed initially: shortest


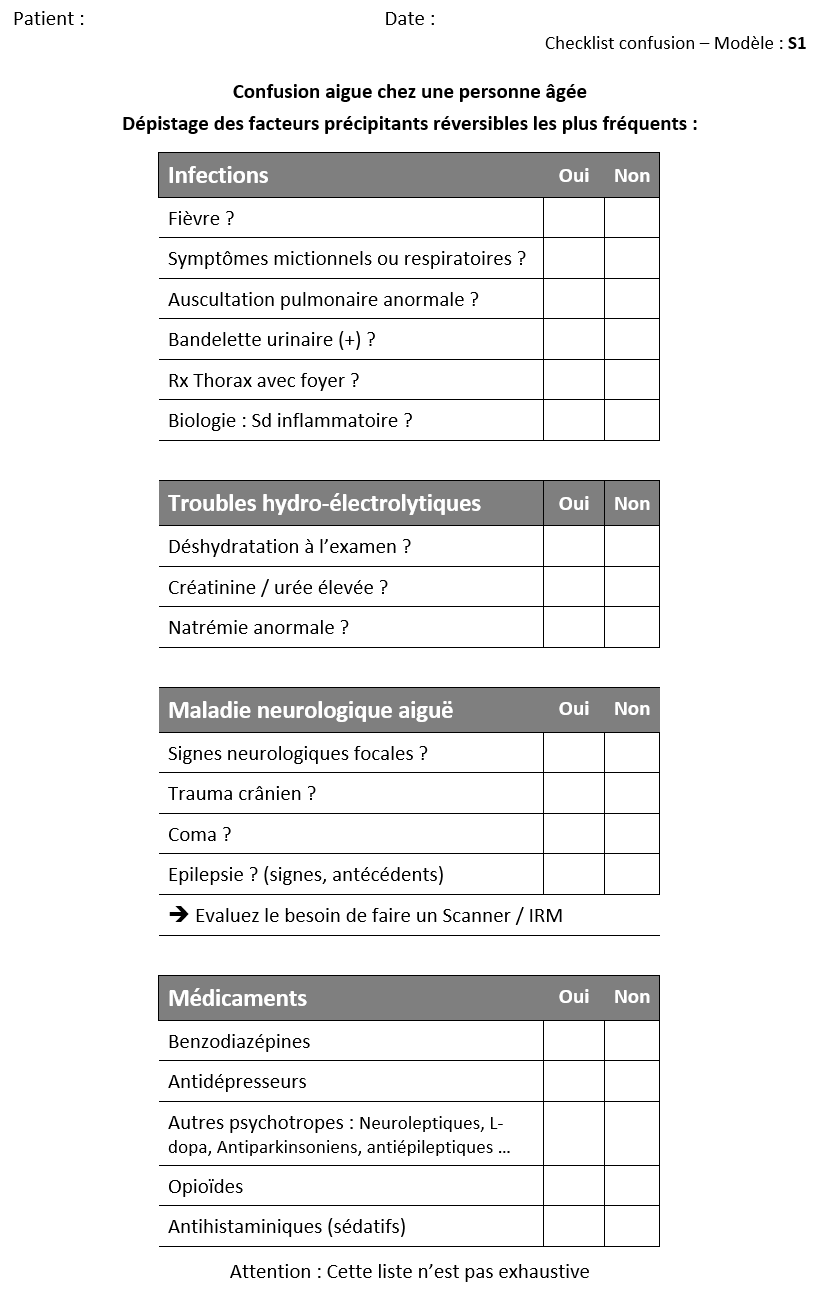


# Figure S2. Draft base models developed initially: medium


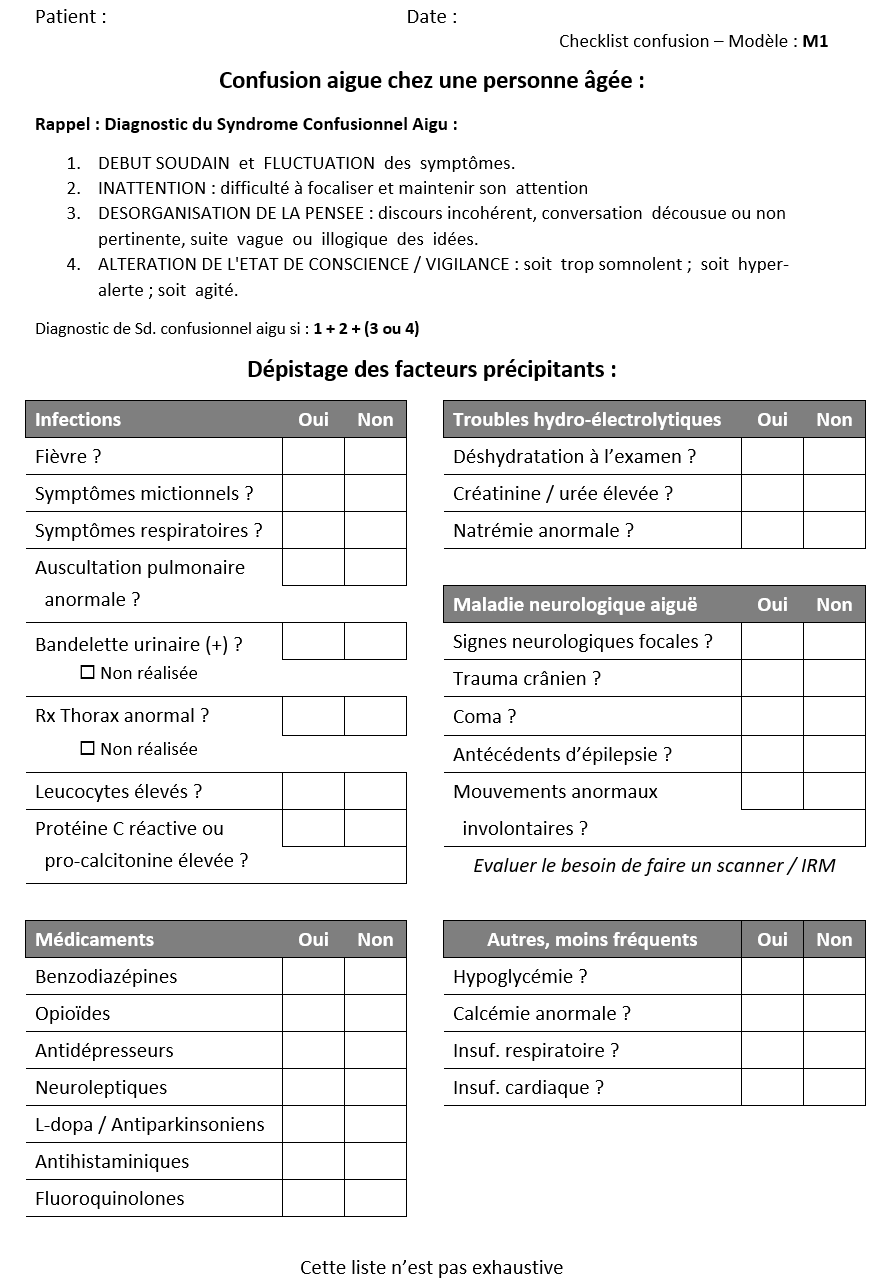


# Figure S3. Draft base models developed initially: longest


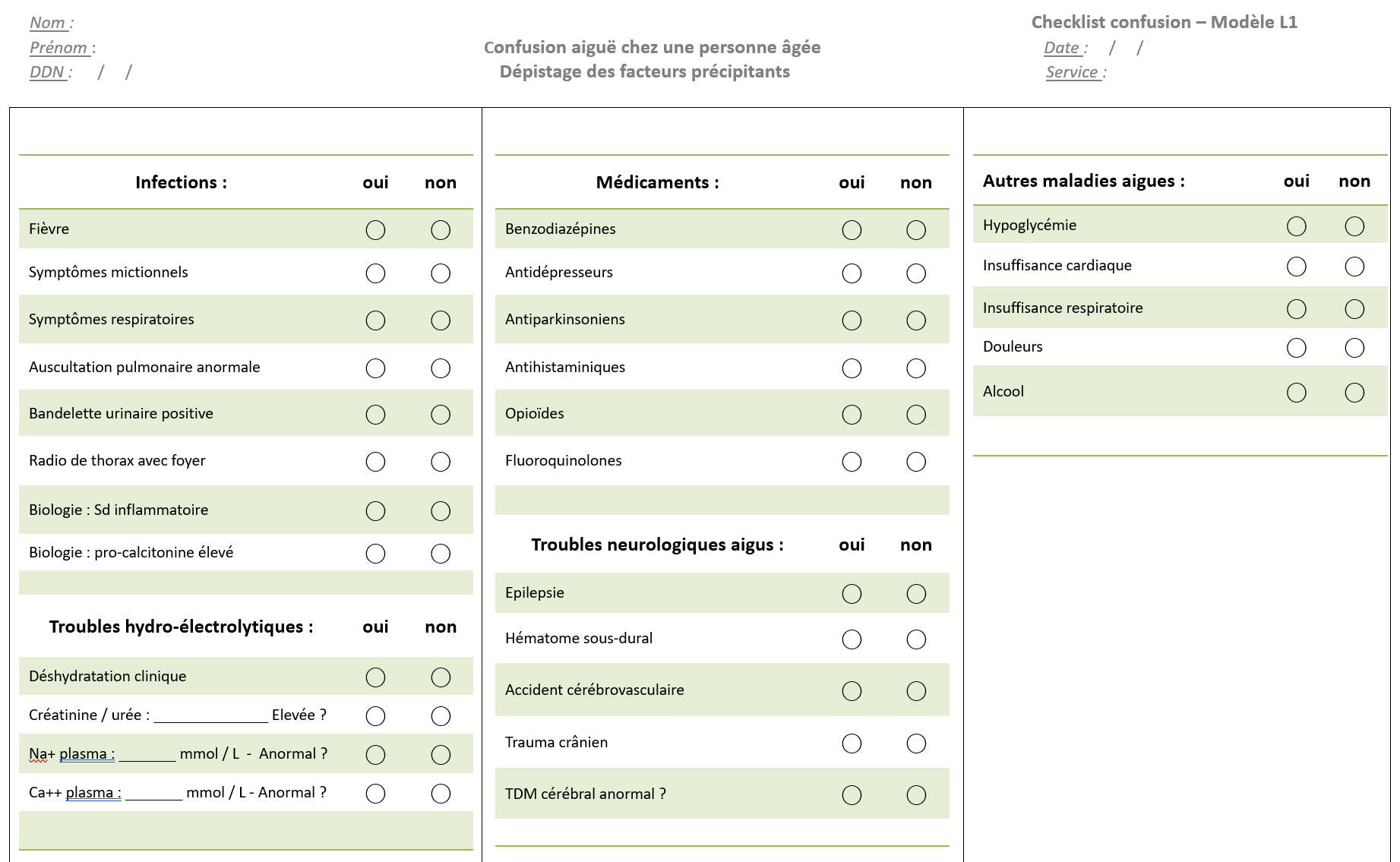


# Figure S4. Final checklist: English and French versions

| \| **Infections:** \| \| **yes** \| **no** \| \| --- \| --- \| --- \| --- \| \| Fever \| \| ⃝ \| ⃝ \| \| Urinary symptoms \| \| ⃝ \| ⃝ \| \| Cough, sputum, dyspnea \| \| ⃝ \| ⃝ \| \| Abnormal pulmonary auscultation \| \| ⃝ \| ⃝ \| \| Abdominal pain \| \| ⃝ \| ⃝ \| \| Urine dipstick: leukocyte+ /nitrite+ \| Not done  ⃝ \| ⃝ \| ⃝ \| \| Biology: increased inflammatory markers \| Not done  ⃝ \| ⃝ \| ⃝ \| \| Lung imaging: consolidation \| Not done  ⃝ \| ⃝ \| ⃝ \| \| **Hydro-electrolytic disorders:** \| \| **yes** \| **no** \| \| Clinically dehydrated \| \| ⃝ \| ⃝ \| \| Abnormal creatinine / urea \| Not done  ⃝ \| ⃝ \| ⃝ \| \| Abnormal natremia \| Not done  ⃝ \| ⃝ \| ⃝ \| \| Abnormal glycemia \| Not done  ⃝ \| ⃝ \| ⃝ \| | \| **Drugs:**  (Recent introduction or change) \| **yes** \| **no** \| \| --- \| --- \| --- \| \| Benzodiazepines and related drugs \| ⃝ \| ⃝ \| \| Antipsychotics (Neuroleptics / atypical) \| ⃝ \| ⃝ \| \| Antidepressants \| ⃝ \| ⃝ \| \| L-dopa / Anti-Parkinson drugs \| ⃝ \| ⃝ \| \| Antihistaminics \| ⃝ \| ⃝ \| \| Opioids (tramadol, codeine, morphine, oxycodone …) \| ⃝ \| ⃝ \| \| Fluoroquinolones \| ⃝ \| ⃝ \| \|  \|  \|  \| \| **Other acute conditions:** \| **yes** \| **no** \| \| Acute heart failure \| ⃝ \| ⃝ \| \| Acute respiratory failure \| ⃝ \| ⃝ \| \| Pain \| ⃝ \| ⃝ \| \| Alcohol (acute intox, withdrawal) \| ⃝ \| ⃝ \| | \| **Acute neurologic disorders:** \| **yes** \| **no** \| \| --- \| --- \| --- \| \| Focal neurologic signs \| ⃝ \| ⃝ \| \| Head trauma \| ⃝ \| ⃝ \| \| History of epilepsy \| ⃝ \| ⃝ \| \| Abnormal involuntary movements \| ⃝ \| ⃝ \| \| - *Is urgent brain CT / MRI required?* \| \| \|     NOTE: This checklist is not exhaustive. Additional, less frequent, causes of delirium may exist. |
| --- | --- | --- | --- | --- | --- | --- | --- | --- | --- | --- | --- | --- | --- | --- | --- | --- | --- | --- | --- | --- | --- | --- | --- | --- | --- | --- | --- | --- | --- | --- | --- | --- | --- | --- | --- | --- | --- | --- | --- | --- | --- | --- | --- | --- | --- | --- | --- | --- | --- | --- | --- | --- | --- | --- | --- | --- | --- | --- | --- | --- | --- | --- | --- | --- | --- | --- | --- | --- | --- | --- | --- | --- | --- | --- | --- | --- | --- | --- | --- | --- | --- | --- | --- | --- | --- | --- | --- | --- | --- | --- | --- | --- | --- | --- | --- | --- | --- | --- | --- | --- | --- | --- | --- | --- | --- | --- | --- | --- | --- | --- | --- | --- | --- | --- | --- | --- | --- | --- |

**For every precipitant found 🡺 Initiate rapidly appropriate treatment**

| REMINDER:  **Think about delirium** in a patient with:   - temporospatial **disorientation,** - acute **change in his/her usual behavior**, - excessive **agitation or drowsiness**, or - inversion of the nychthemeral rhythm   **Diagnostic criteria ***  1- ACUTE ONSET or fluctuating symptoms.  2- INATTENTION: Do the patient have difficulty focusing and maintaining attention?  3- DISORGANIZED THINKING: incoherent thinking, rambling or irrelevant conversation, unclear or illogical flow of ideas.  4- ALTERED LEVEL OF CONCIOUSNESS: either lethargic, in a stupor, or hyperalert, or agitated.  Delirium diagnosed if: **1 + 2 + (3 or 4)**  * *Confusion Assessment Method (CAM)* | INFORMATION:  **Most frequent precipitant factors:**  - Infections (49-69 % of cases, depending on studies):   - Urinary tract infection - Lung infection - Systemic inflammatory response / Sepsis   - Hydro-electrolytic disorders (32 - 46%) :   - Dehydration - Hypo- or hypernatremia - Hypercalcemia   - Drugs (31 - 47%) :   - Psychotropic drugs: benzodiazepines, antidepressants, antipsychotics - Opioids - Fluoroquinolones   - Acute neurological disorders (11 - 18%) :   - Epilepsy - Subdural hematoma - Stroke - Meningitis / Encephalitis - Traumatic brain injury   - Other acute conditions (14 - 38%) :   - Heart failure - Hypoglycemia / diabetic coma - Respiratory failure - Alcohol / Illicit drugs   - Frequently associated with delirium :   - Pain - Urinary retention - Fecalome   Warning: this list is not exhaustive | INFORMATION:  **Frequent pre-existing predisposing factors:**   - Known dementia or pre-existing cognitive impairment (26 - 74% of patients, depending on studies) - History of neurological disease (24 - 33%) : stroke, Parkinson, epilepsy … - History of psychiatric condition (25 - 30%) : depression, psychosis, anxiety … - Visual or hearing impairment, mild (12 to 25%) or severe (7 to 10%) |
| --- | --- | --- |

| \| **Infections :** \| \| **oui** \| **non** \| \| --- \| --- \| --- \| --- \| \| Fièvre \| \| ⃝ \| ⃝ \| \| Symptômes mictionnels \| \| ⃝ \| ⃝ \| \| Toux, dyspnée, crachats \| \| ⃝ \| ⃝ \| \| Auscultation pulmonaire anormale \| \| ⃝ \| ⃝ \| \| Douleurs abdominales \|  \| ⃝ \| ⃝ \| \| Bandelette urinaire : positive \| Non fait  ⃝ \| ⃝ \| ⃝ \| \| Biologie : Sd. inflammatoire \| Non fait  ⃝ \| ⃝ \| ⃝ \| \| Imagerie pulmonaire : foyer \| Non fait  ⃝ \| ⃝ \| ⃝ \| \| **Troubles hydro-électrolytiques :** \| \| **oui** \| **non** \| \| Déshydratation clinique \| \| ⃝ \| ⃝ \| \| Créatinine / urée anormale \| Non fait  ⃝ \| ⃝ \| ⃝ \| \| Na+ (sang) anormal \| Non fait  ⃝ \| ⃝ \| ⃝ \| \| Glycémie (capillaire) anormale \| Non fait  ⃝ \| ⃝ \| ⃝ \| | \| **Médicaments :**  (introduction ou modification récente) \| **oui** \| **non** \| \| --- \| --- \| --- \| \| Benzodiazépines et apparentés \| ⃝ \| ⃝ \| \| Antipsychotiques (Neuroleptiques ou atypiques) \| ⃝ \| ⃝ \| \| Antidépresseurs \| ⃝ \| ⃝ \| \| L-dopa / Antiparkinsoniens \| ⃝ \| ⃝ \| \| Antihistaminiques \| ⃝ \| ⃝ \| \| Opioïdes (tramadol, codéine, morphine, oxycodone …) \| ⃝ \| ⃝ \| \| Fluoroquinolones \| ⃝ \| ⃝ \| \|  \|  \|  \| \| **Autres situations aigues :** \| **oui** \| **non** \| \| Insuffisance cardiaque aigue \| ⃝ \| ⃝ \| \| Insuffisance respiratoire aigue \| ⃝ \| ⃝ \| \| Douleurs \| ⃝ \| ⃝ \| \| Alcool (intox aigue, sevrage) \| ⃝ \| ⃝ \| | \| **Affections neurologiques aiguës :** \| **oui** \| **non** \| \| --- \| --- \| --- \| \| Signes neurologiques focaux \| ⃝ \| ⃝ \| \| Trauma crânien \| ⃝ \| ⃝ \| \| Antécédents d’épilepsie \| ⃝ \| ⃝ \| \| Mouvements anormaux involontaires \| ⃝ \| ⃝ \| \| - *Faut-il un scanner / IRM cérébral urgent ?* \| \| \|     NOTE : cette checklist n’est pas exhaustive. Ils existent d’autres causes, moins fréquentes, de confusion aigue. |
| --- | --- | --- | --- | --- | --- | --- | --- | --- | --- | --- | --- | --- | --- | --- | --- | --- | --- | --- | --- | --- | --- | --- | --- | --- | --- | --- | --- | --- | --- | --- | --- | --- | --- | --- | --- | --- | --- | --- | --- | --- | --- | --- | --- | --- | --- | --- | --- | --- | --- | --- | --- | --- | --- | --- | --- | --- | --- | --- | --- | --- | --- | --- | --- | --- | --- | --- | --- | --- | --- | --- | --- | --- | --- | --- | --- | --- | --- | --- | --- | --- | --- | --- | --- | --- | --- | --- | --- | --- | --- | --- | --- | --- | --- | --- | --- | --- | --- | --- | --- | --- | --- | --- | --- | --- | --- | --- | --- | --- | --- | --- | --- | --- | --- | --- | --- | --- | --- | --- |

**Pour tout précipitant retrouvée 🡺 Démarrez rapidement un traitement adapté**

| RAPPEL :  **Penser à une Confusion mentale** (Sd. confusionnel aigu) devant :   - une **désorientation** temporo-spatiale, - un **changement aigu du son comportement** habituel, - une **agitation ou somnolence** excessive, ou - une inversion du rythme nycthéméral   **Critères diagnostiques ***  1-DEBUT AIGU et fluctuation des symptômes  2-INATTENTION : est-ce que le patient a de la difficulté à focaliser et maintenir son attention ?  3-DESORGANISATION DE LA PENSEE : discours incohérent, conversation décousue ou non pertinente, suite vague ou illogique des idées.  4- ALTERATION DE L'ETAT DE CONSCIENCE / VIGILANCE : soit trop somnolent ; soit hyper-alerte ; soit agité.  Diagnostic de Sd. confusionnel aigu si : **1 + 2 + (3 ou 4)**  * *Confusion Assessment Method (CAM)* | POUR INFO :  **Précipitants aigus les plus fréquents :**  - Infections (49 - 69 % des cas, selon les études) :   - Infection urinaire - Infection pulmonaire - Sd infectieux sans foyer évident / Sepsis   - Troubles hydro-électrolytiques (32 - 46%) :   - Déshydratation - Hypo- ou hypernatrémie - Hypercalcémie   - Médicaments (31 - 47%) :   - Psychotropes : benzodiazépines, antidépresseurs, antipsychotiques - Opioïdes - Fluoroquinolones   - Pathologies neurologiques aigues (11 - 18%) :   - Epilepsie - Hématome sous-dural - Accident cérébrovasculaire - Méningite / Encéphalite - Trauma crânien   - Autres situations aigues (14 - 38%) :   - Insuffisance cardiaque - Hypoglycémie / coma diabétique - Insuffisance respiratoire - Alcool / drogues   - Souvent associés au sd confusionnel :   - Douleur - Rétention urinaire - Fécalome   Attention : cette liste n’est pas exhaustive | POUR INFO :  **Facteurs prédisposants préexistants fréquents :**   - Trouble cognitif majeur avéré ou troubles cognitifs chroniques sous-jacents non étudiés (26 - 74% des patients, selon les études) - Antécédents de maladie neurologique (24 - 33%) : maladie cérébrovasculaire, Parkinson, épilepsie … - Antécédents de maladie psychiatrique (25 - 30%) : dépression, psychose, sd. anxieux … - Déficit visuel ou auditif, modéré (12 à 25%) ou sévère (7 à 10%) |
| --- | --- | --- |
